# Systematic review of associations between gut microbiome composition and stunting in under-five children

**DOI:** 10.1038/s41522-024-00517-5

**Published:** 2024-05-23

**Authors:** Mwelwa Chibuye, Daniel R. Mende, Rene Spijker, Michelo Simuyandi, Chaluma C. Luchen, Samuel Bosomprah, Roma Chilengi, Constance Schultsz, Vanessa C. Harris

**Affiliations:** 1 Department of Global Health, Amsterdam Institute for Global Health and Development (AIGHD), Amsterdam University Medical Centers, University of Amsterdam, Amsterdam, The Netherlands; 2 Research Division, Centre for Infectious Disease Research in Zambia, Lusaka, Zambia; 3 Amsterdam Institute of Infection and Immunity, Amsterdam University Medical Centers, Amsterdam, The Netherlands; 4 Department of Medical Microbiology and Infection Control, Amsterdam University Medical Centers, Amsterdam, The Netherlands; 5 Department of Biostatistics, School of Public Health, University of Ghana, Legon, Accra, Ghana; 6 The Zambia National Public Health Institute (ZNPHI), Lusaka, Zambia; 7 Division of Infectious Diseases, Department of Internal Medicine, Amsterdam University Medical Centers, University of Amsterdam, Amsterdam, The Netherlands

**Keywords:** Health care, Microbiome, Clinical microbiology, Policy and public health in microbiology

## Abstract

Childhood stunting is associated with impaired cognitive development and increased risk of infections, morbidity, and mortality. The composition of the enteric microbiota may contribute to the pathogenesis of stunting. We systematically reviewed and synthesized data from studies using high-throughput genomic sequencing methods to characterize the gut microbiome in stunted versus non-stunted children under 5 years in LMICs. We included 14 studies from Asia, Africa, and South America. Most studies did not report any significant differences in the alpha diversity, while a significantly higher beta diversity was observed in stunted children in four out of seven studies that reported beta diversity. At the phylum level, inconsistent associations with stunting were observed for Bacillota, Pseudomonadota, and Bacteroidota phyla. No single genus was associated with stunted children across all 14 studies, and some associations were incongruent by specific genera. Nonetheless, stunting was associated with an abundance of pathobionts that could drive inflammation, such as *Escherichia/Shigella* and *Campylobacter*, and a reduction of butyrate producers, including *Faecalibacterium, Megasphera, Blautia*, and increased *Ruminoccoccus*. An abundance of taxa thought to originate in the oropharynx was also reported in duodenal and fecal samples of stunted children, while metabolic pathways, including purine and pyrimidine biosynthesis, vitamin B biosynthesis, and carbohydrate and amino acid degradation pathways, predicted linear growth. Current studies show that stunted children can have distinct microbial patterns compared to non-stunted children, which could contribute to the pathogenesis of stunting.

## Introduction

Stunting is the most prevalent form of childhood malnutrition, affecting approximately 22% or 148.1 million children under five years old in 2022^1^. Defined by the World Health Organization (WHO) as impaired growth and development experienced in children due to inadequate nutrition and repeated infections, it is often reported as a height for age more than two standard deviations below the standard child growth median. Although the global prevalence of childhood stunting has decreased from 30.1 to 22.2% in the past twenty years, there have been minimal reductions in the highest-burden regions of Africa and South Asia, where 42% and 53%, respectively, of all stunted children, live^1,2^. As the population in low- and middle-income countries (LMICs) continues to grow, the number of children affected by stunting will likely increase. Stunting has a range of adverse outcomes, including increased susceptibility, incidence, and severity of infectious diseases (particularly pneumonia and diarrhea), poor cognitive development, and mortality^3–5^. The pathophysiology of childhood stunting is likely multifaceted. Poor sanitation, recurrent infections, poor nutrition, and genetic predisposition are traditionally implicated in stunting^6–8^. However, despite the epidemiological and statistical associations, the pathophysiology of stunting remains unclear. Recent studies have pointed to the role of the gut microbiome in normal childhood growth and that alterations in the gut microbiome composition are associated with stunting and may play a role in its development^9,10^.

The gut microbiome, a complex community of bacteria, eukaryotic and prokaryotic viruses, fungi, and archaea in the gastrointestinal tract, has a range of functions relevant to normal childhood growth and development. These include the metabolism of macro- and micro-nutrients, production of vitamins for growth and maintaining gut health, hormone regulation facilitating production and energy harvesting from nutrients, and helping maintain the intestinal mucosa’s structure and barrier function^11–15^. The gut microbiome also shapes the development of the innate and adaptive immune system and provides colonization resistance against exogenous pathogens and enteric infections^16–18^. The colonization of the intestinal microbiome overlaps with critical developmental periods of child growth. Young children, particularly in LMICs, are at high risk for microbiota perturbation secondary to frequent infectious illness episodes, antibiotic use, poor nutrition, and unsanitary living environments. However, how these perturbations, and the composition and function of the developing microbiome, may translate into growth outcomes and health is uncertain^9,19–21^.

Although multiple studies have evaluated correlations between intestinal microbiome composition and stunting, and several review articles have been written on this subject, no systematic reviews have systematically evaluated and synthesized the associations between microbiome composition and stunting outcomes. Given the therapeutic potential of the microbiome and the need to understand its potential etiologic role in the development of stunting, this systematic review aimed to summarize and evaluate the existing literature that correlates the composition of the gut microbiota and childhood stunting in LMICs.

## Results

### Study summary overview

#### Study selection

**Fig. 1:**
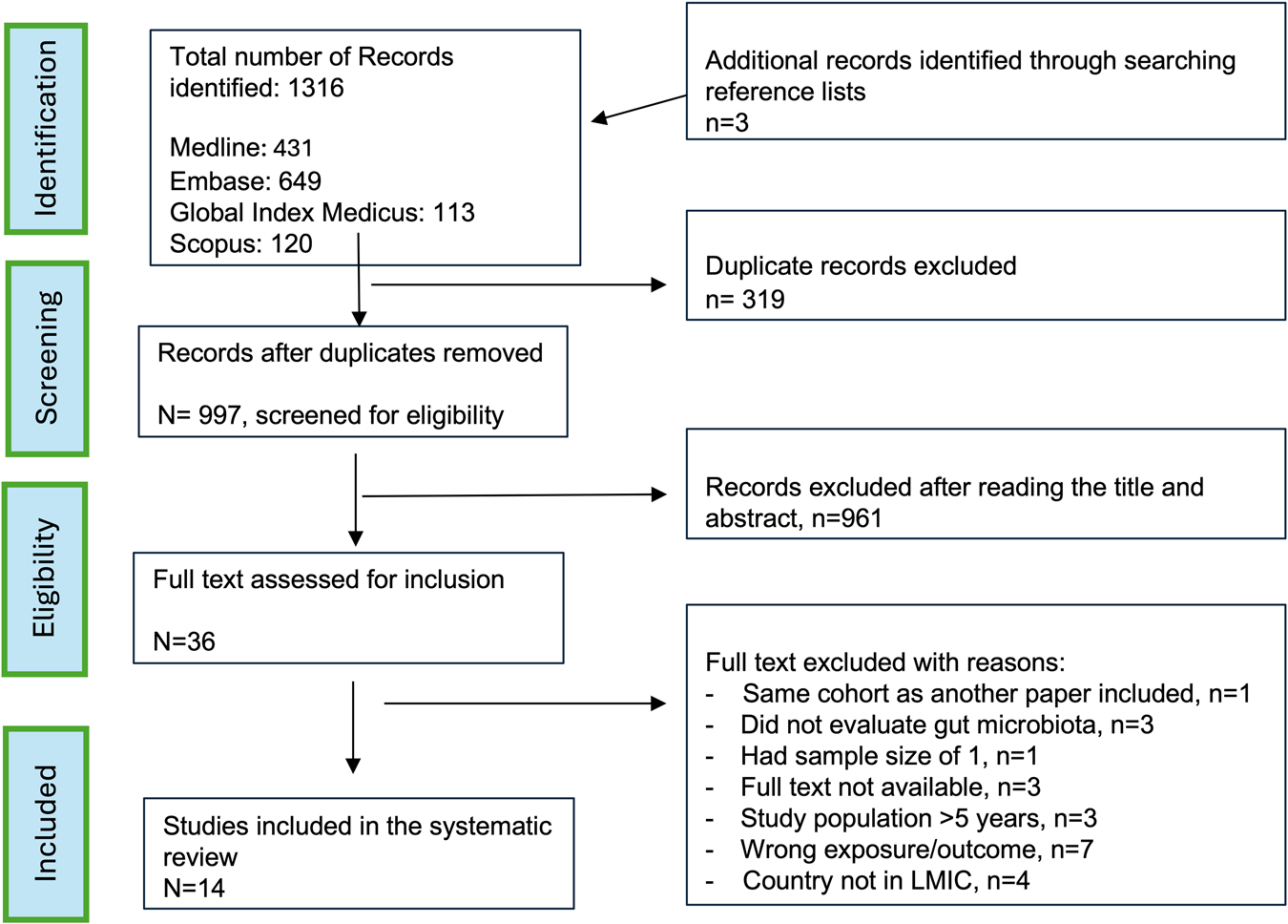
PRISMA flow chart for selection of articles.

The literature search identified 935 studies (Medline-431, Embase-625, SCOPUS-120, and the Global Index Medicus-113). Three studies were identified by searching reference lists and grey literature. After removing duplicates and screening using titles and abstracts for inclusion and exclusion criteria, 39 articles were selected for full-text reading, and 14 articles met all the criteria and are included in this review. Figure 1 shows the PRISMA flow chart of article screening and selection stages.

#### Summary of included studies

Of the fourteen included studies, eight were conducted in Asia (Bangladesh^22–25^, India^26,27^, Indonesia^28,29^, five in Africa (Malawi^25,30,31^, Zimbabwe^32^ Madagascar, and the Central African Republic^33^ and two in Peru, South America^34,35^. Six studies assessed child-level microbiome characteristics at one time point: four were cross-sectional studies^27,28,33^, and two were case-control studies^22,23^. Eight studies evaluated the gut microbiome over a period of time: five^24–26,29,30^ assessed the microbiome in stunted children at baseline, and another followed up time point, while three studies^31,34,35^ followed healthy children and compared the gut microbiome in those who developed stunting with those who did not. The age of the children in the study population varied between studies from birth (0 days) to 5 years, while the number of study participants ranged from 20 to 404. A summary of the included studies is shown in Table 1.

**Table 1:** Summary characteristics of the included studies

First Author, Year	Study location and year	Study design /study duration	Age, sample size	Sample type and sampling times	Outcome definition	Sequencing platform/ Target region	Summary findings	Quality score (JBI)*
**ASIA**								
Shivakumar et al.^30^	India, 2017	cross-sectional,	18–24 months, 41 non-breastfeeding participants (16 stunted, 25 non-stunted)	Stool collected at enrolment	LAZ < −2	16S rDNA V3-V4 region Seq: Illumina Pipeline: QIIME 2	Higher abundance of Prevotella and Sutterella and less Clostridium and Bifidobacterium in Stunted children	**6/8**
Dinh et al.^29^	India, 2009–2010	Case-control, 2 years	birth cohort(0–3yrs), 20 participants (10 persistently stunted and 10 non-stunted)	Stool collected every 3 months from 3 to 24 months of age	Persistent stunting (LAZ<-3) at 6 to 8 timepoints	16S rRNA V4 region Seq: Illumina MiSeq Pipeline: QIIME Version 1.8	Higher abundance of Desulfovibrio genus and Campylobacterales order in stunted children	**8/10**
Surono et al.^31^	Indonesia, 2020	cross-sectional	3–5 years, 131 participants (78 stunted and 53 non-stunted)	Stool collected at enrolment	stunted, severely stunted (LAZ < −2, LAZ < −3)	16S rRNA V3-V4 region Seq: Illumina MiSeq Pipeline: QIIME 2	14 taxa-Higher abundance of *Prevotella* 9, *Bacteroides, Alloprevotella*, *Senegalimassilia*, *Butyrivibrio*, Family XIII AD3011 group, *Coprococcus* 3 *Ruminococcaceae* UCG-010, an uncultured genus of Erysipelotrichaceae and 2 uncharacterized taxa, and low abundance of *Coprobacter*, *Lachnospira, Enterobacter, an* unidentified genus from *Enterobacteriaceae* in stunted children	**6/8**
Chen et al.^25^	Bangladesh, 2016–2018	case-control	12–18 months, 137 participants (110 stunted and 27 non-stunted)	Stool, gastric and duodenal samples collected at enrolment	LAZ <-2	16S rDNA V4 region Seq: Illumina NextSeq 500 Pipeline: DESeq2	14 duodenal taxa (*Veilonella* sp., *Streptococcus* sp., *Gemella* sp., *Johnsonella* sp., *Rothia mucilaginosa, Neisseria subflava, Actinomyces* sp., *Haemophilus* sp., *Granulicatella elegans, Corynebacterium* sp., *Prevotella melaninogenica, Fusobacterium* sp., *Leptotrichia* sp., *Leptotrichia shahii)* were significantly higher in stunted children	**8/10**
Khan Mirzaei et al.^26^	Bangladesh, 2016–2017	case-control, 18 months	14–38 months, 60 participants (30 stunted, 30 non-stunted)	Stool collected at baseline and 18m time point	HAZ<-2	16S rRNA gene and shotgun sequencing Seq: Illumina MiSeq Pipeline: DADA2 & QIIME2	Higher abundance of Pseudomonoadota, *Escherichia coli* and *Klebsiella* in stunted children	**6/10**
Masrul et al.^32^	Indonesia, Year not reported	case-control	Children ≤3 years, 96 children (48 stunted and 48 non-stunted)	Stool collected at enrolment	HAZ<-2	16S rRNA V3-V4 Seq: Illumina MiSeq platform Pipeline: QIIME	61 species of bacteria identified only in stunted children.	**6/10**
Perin et al.^27^	Bangladesh, 2014–2015	case-control, 18 months	6–31 months, 68 children	Stool collected at enrolment and 18 months later	negative ΔHAZ over 18 months	16S rRNA V1–V3 region Seq: Illumina® MiSeq Pipeline: DADA2	Stunted children had more Pseudomonoadota (*Escherichia/Shigella*) and decreased abundance of Prevotella	**10/10**
Gough et al.^28^	Malawi, Bangladesh Year not reported	Case-control i) Malawi- median 9.7 months ii) Bangladesh-median 14.5 months	Twin cohorts from i) Malawi: 18 participants (10 severe stunted, 8 control) ii) Bangladesh: 11 participants (6 severe stunted, 5 control- Bangladesh	Stool collected up to 8 collection time points	HAZ<-2	Seq: Shotgun whole genome sequence Pipeline: NR	*Acidaminococcus* sp. At baseline was significantly negatively associated with stunting (HAZ Zscores)	**7/10**
**AFRICA**								
Desai et.al.^33^	Malawi, Year not reported	Prospective cohort, 6 months	12 to 43 months, 49 children without diarrheaor HIV	Stool collected at enrolment, 3 months and 6 months later,	ΔHAZ <−0.3	16S rRNA V4 region (F515/R806) Seq: Illumina MiSeq Pipelines: PhyloSeq (DESeq2) and other R packages	10 taxa (*Clostridium XIVa, Olsenella, Megasphera NA, Providencia NA, Prevotella NA (two), Bifidobacterium ruminantium, Helicobacter fennelliae, Bacteroides dorei, and Parabacteroides merdae)* were absent in stunted children.	**8/10**
Kamng’ona et al.^34^	Malawi Year not reported	Prospective cohort, One year	6 months, 691 children	Stool collected at 6, 12, and 18 months,	A negative change in LAZ	16S rRNA V4 region Seq: Illumina MiSeq Pipeline: NR	No taxa were significantly different in stunted and non-stunted children	**6/10**
Vonaesch et al.^36^	Central African Republic (CAR.) and Madagascar, 2016–2017	cross-sectional	2–5 years, 404 participants (74 severely stunted, 94 moderately stunted, 236 controls)	Stool and duodenal samples collected at one time point	severe (HAZ≤ −3), moderate (HAZ >=−3, ≤ HAZ<−2 and no stunting (HAZ>−2)	16S rRNA V4 region Seq: MiSeq Pipeline: QIIME v1.9	Oropharyngeal taxa (including *Veillonella, Gemella, Streptococcus, Neisseria, Fusobacterium, Actinobacillus, and Porphyromonas)*, enteropathogens *(Escherichia coli/Shigella sp. And Campylobacter sp.)*, and reduced *Clostridium* were significantly negatively associated with stunting (LAZ Zscores)	6/8
Robertson et al.^35^	Zimbabwe	Prospective cohort, 18 months	1 month, 385 participants	Stool samples collected a mean of 2.6 timepoints	LAZ<-2	Metagenomics Illumina HiSeq 2500 Pipeline: MetaPhlAn3	No significant differences were observed in the taxa of stunted and non-stunted children. Metagenomic pathways were better predictors of linear growth.	
**SOUTH AMERICA**								
Rouhani et. al.^37^	Peru Year not reported	Retrospective birth cohort, 2 years	0–17 days, 271 children	Stool collected at 6, 12, 18, and 24 months	stunted, severely stunted (LAZ < −2, LAZ < −3)	16S rDNA V4 region Seq: NR Pipeline: DADA2	Campylobacter was significantly negatively associated with stunting (low LAZ)	**8/10**
Zambruni, et al.^38^	Peru, Dec 2014 to May 2015	prospective cohort, 6 months	5–12 months, 78 non-stunted children	Stool collected at baseline and 6 months later	LAZ<-2	16S, V4 region Seq: Illumina MiSeq sequence Pipeline: QIIME and Mothur	*Ruminococcus* and *Collinsella* increased over time only in children who became stunted	**7/10**

*JBI quality score ranks the risk of bias: ≤4.9 = high risk of bias, 5.0-6.9 = moderate risk of bias, above 7.0 = low risk of bias.

The gut microbiome was assessed in fecal samples for all studies. In addition to fecal sampling, the gut microbiota was assessed using duodenal^22,33^ and gastric samples^33^. Except for two studies that used shotgun sequencing^23,25^, all studies evaluated the gut microbiome using 16S rRNA sequencing, although the region of amplification differed between the studies: V4 (*n* = 7), V3-V4 (*n* = 3), and V1-V3 (*n* = 1). Stunting in most studies was defined stunting using the WHO-standard definition of HAZ/LAZ z-score <−2. Three studies described stunting as poor growth velocity defined as a negative change in LAZ scores between two time points, specifically a difference of less than −0.3 between two growth measurement points^30^ and any negative change in HAZ/LAZ^24,31^.

#### Quality and Risk of Bias Assessment

All studies were evaluated for methodological quality using the Joanna Briggs Institute (JBI) critical appraisal tool. The largest risk of bias in almost all studies was related to unassessed potential confounding, as most studies did not adjust for confounders, such as diet and antibiotics use that could significantly influence the gut microbiome. Overall, the risk of bias for the studies was categorized as low or moderate (Supplementary Table 2).

### Primary Outcomes

#### Alpha and beta diversity in stunted and non-stunted children

**Fig. 2:**
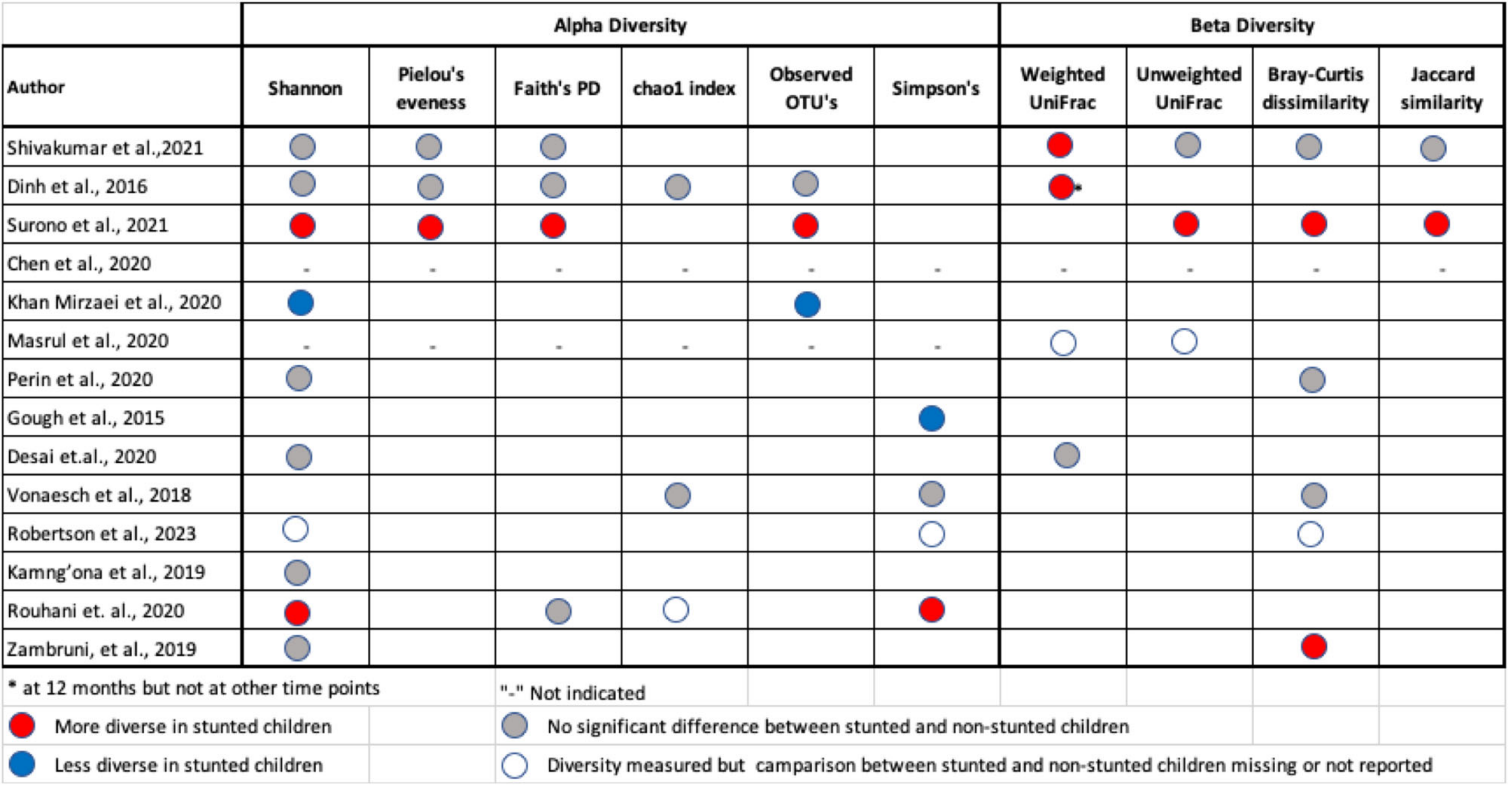
Summary comparison of alpha and beta diversity assessment between stunted and non-stunted children.

Of the included studies, 12 reported alpha diversity in stunted children using varying alpha diversity indices (Fig. 2). Only one study reported a higher alpha diversity in stunted children (using four diversity indices: Shannon, Pielou’s evenness, observed taxonomic units (OTUs), and Faith’s PD)^28^. Six studies reported a similar alpha diversity (Shannon) for stunted and non-stunted children^24,26,27,30,31,35^, and two studies using metagenomic sequencing methods reported significantly lower alpha diversity for Simpson’s^25^, Shannon’s and OTU’s^23^ indices. Four studies^22,29,32,34^ did not report alpha diversity.

**Fig. 3:**
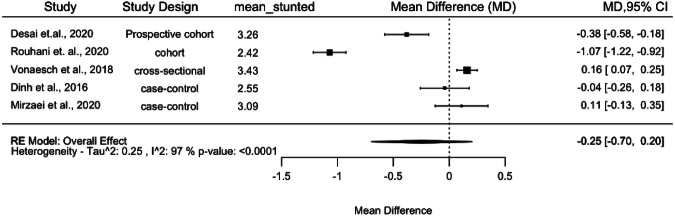
Forest plot of the differences in alpha diversity between stunted and non-stunted children by Shannon index.

Of the 14 studies included in this review, five studies^23,26,30,33,34^ had publicly available sequence and metadata available to re-assess for alpha diversity. We conducted a meta-analysis using the random effects model to test the association between stunting and Shannon diversity from publicly available raw reads. The forest plot shows that substantial heterogeneity was observed (I^2^=97%; *p* < 0.001), with no significant differences in Shannon diversity between stunted and non-stunted children (Fig. 3). We did not perform a metadata analysis due to the heterogeneity observed among the studies.

Similarly, the results of between-group comparisons for beta diversity were inconsistent among the studies (Fig. 2). Four studies reported a higher beta diversity in stunted children using weighted uniFrac distance^26,27^, unweighted uniFrac^28^, and Bray-Curtis dissimilarity^28,35^. No significant differences were observed between stunted and non-stunted children in four studies when using different measures: weighted uniFrac distance^30^, unweighted uniFrac^27^, and Bray-Curtis dissimilarity^24,27,33^. Seven studies did not report on beta diversity.

#### Differences in microbial composition at the phylum level

Six studies reported differences in microbial composition at the phylum level in stunted and non-stunted children; all were conducted in Asia (Table 2). Stunted children had a higher abundance of Bacillota^28,29^, Bacteroidota^26,29^, and Pseudomonoadota^23,29^. A lower abundance for Bacteroidota was reported in stunted children in^28^, while no significant differences were observed between stunted and non-stunted children for Pseudomonoadota^24,26,28^; Bacteroidota^24^; Bacillota^23,24,26^; and Actinomycetota^23,24,26,28^.

**Table 2:** Phylum-level differences in microbial composition between stunted and non-stunted children

Study (Author, Year)	Bacillota	Bacteroidota	Pseudomonoadota	Actinomycetota
**Shivakumar et al., 2021** Sample size: 41 Age: 18–24 months	NS	NS	NS	NS
**Dinh et al., 2016** Sample size: 20 Age: 0–3yrs),	NS	Higher in stunted children at 12 m (P = 0.043)	NS	NS
**Surono et al., 2021** Sample size: 131 Age: 3–5 years	Higher in stuntedchildren, 45.7%vs39.8% (p= 5.89*10^−4^)	Lower in stunted, 51.3% (p=2.55*10^−4^),	NS	NS
**Khan Mirzaei et al., 2020** Sample size: 60 Age: 14–38 months	NS	NR	Higher in stunted children. (Sig.not reported)	NS
**Masrul et al., 2020** Sample size: 96 Age: ≤3 years	Higher in stunted children, 47.52% (sig. not reported)	Higher in stunted children, 16.15% (Sig. not reported)	Higher in stunted children, 21.12% (Sig. not reported)	NR
**Perin et al., 2020** Sample size: 68 Age: 6–31 months	NS	NS	NS	NS

Note: “NR ”- data on phylum not reported for the study. “NS”- No significant difference between stunted and non-stunted children. “Sig.”- Significance.

#### Differences in the microbial composition at the genus level

**Fig. 4:**
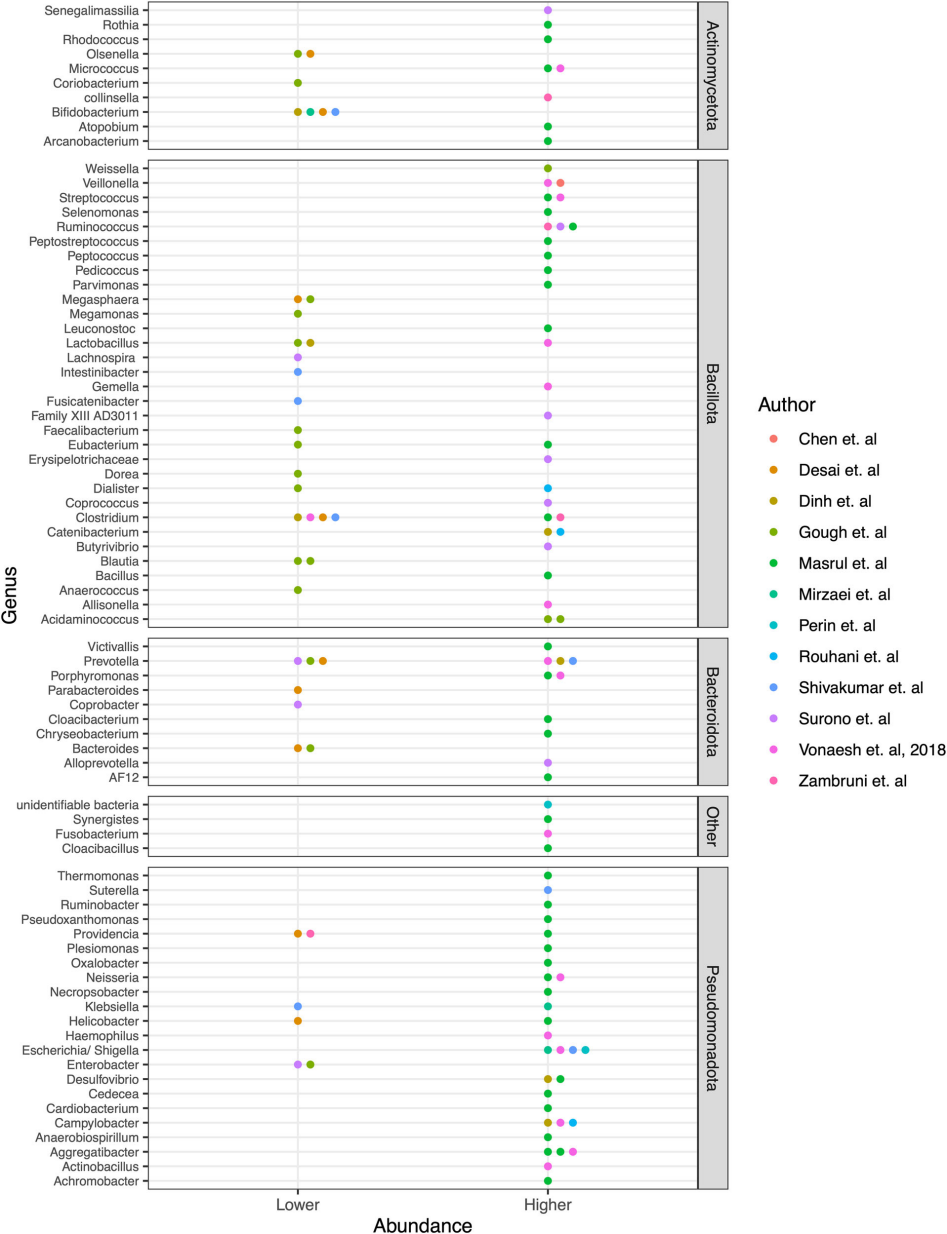
Overview of bacterial genera in fecal samples that have a significantly higher or lower relative abundance in stunted children. “Other phyla” includes Fusobacteriota, Verrucomicrobiota, and Synergistota.

Twelve studies reported significant differences in the relative bacterial abundances of fecal samples in stunted vs. non-stunted children at the genus level (Fig. 4). They report an over-representation of genera from the Baccilota and Pseudomonadota phyla in stunted children. However, associations between these genera and stunting were not always consistent. A higher relative abundance was observed for Bacillota (*Weisella, Veillonella, Allisonella, Acidaminococcus, Catenibacterium, and Streptococcus)*, Pseudomonadota (*Escherichia/Shigella, Desulfovibrio, Aggregatibacter, Neisseria, Campylobacter* and others). Taxa from other phyla in higher abundance in stunted children included *Micrococcus and Rothia* (Actinomycetota) and *Porphyromonas* (Bacteroidota). Taxa with a significantly lower relative abundance in stunted children included Bacillota (*Megasphaera, Dorea, Lachnospira, Megamonas, Blautia, Bacteroides, Enterobacter, Anearococcus)*, Bacteroidota *(Bacteroides, Parabacteroides, Coprobacter)*, Actinomycetota *(Olsenella, Bifidobacterium) and* Pseudomonadota *(Enterobacter)*. Discrepant or inconsistent associations were reported for ten genera: *Providencia, Prevotella, Ruminococcus, Lactobacillus, Klebsiella, Helicobacter, Faecalibacterium, Eubacterium, Dialister, and Clostridium*. Two studies^31,32^ did not report any significant difference in the microbiome composition of stunted and non-stunted children. The complete list of genera identified and their associations with stunting are shown in Fig. 3. We also observed that only *Campylobacter* was reported to be significantly higher in stunted children across the three regions. Stunted children in Africa and Asia had more shared taxa, including an abundance of *Escherichia/Shigella, Aggregatibacter, Veillonella, Streptococcus*, and reduced *Bifidobacterium, Blautia*, and *Megasphera* (Supplementary Fig. 1).

#### Duodenal and gastric microbiota

**Fig. 5:**
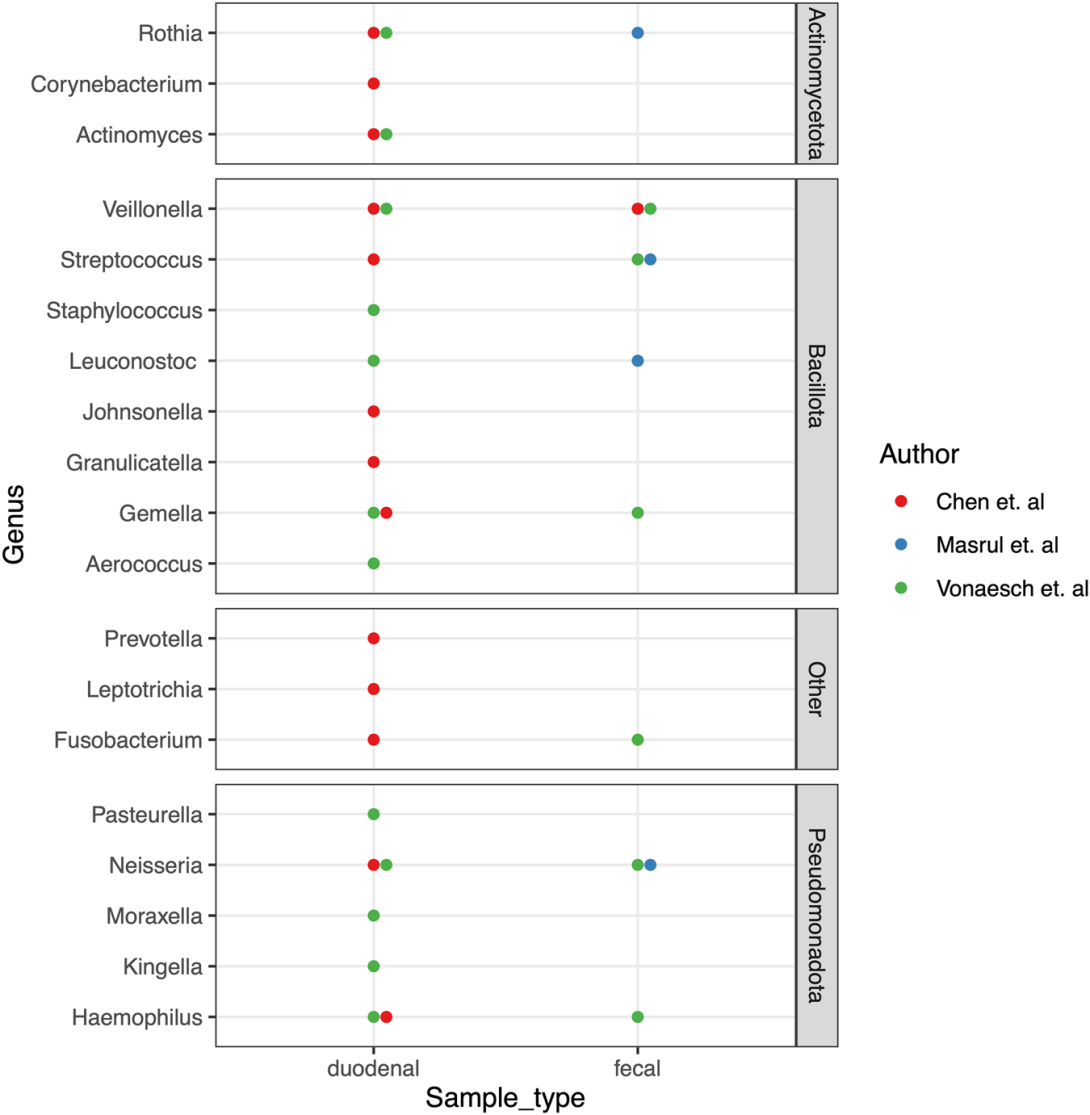
Summary of taxa with significantly higher abundance in duodenal and fecal samples of stunted children at the genus level. “Other phyla” include Bacteroidota and Fusobacteriota.

The duodenal microbiota in stunted was reported in two studies from Asia (Bangladesh)^22^ and Africa (Madagascar and Central African Republic)^33^ (Fig. 5). In both studies, the duodenal samples from stunted children had an overrepresentation of *Veillonella*, *Gemella*, *Neisseria, Actinomyces, Haemophilus*, and *Rothia* species. We cross-referenced taxa from the duodenum with those from stool to assess taxa present in both sample types and found *Rothia, Veillonella, Streptococcus, Gemella, Fusobacterium, Neisseria, and Haemophilus* were abundant in both samples (Fig. 5). However, several taxa with a higher abundance in stunted children were only reported in duodenal samples, including *Staphylococcus, Pastuerella, Johnsenella, Moraxella, and Kingella*. We also observed that other than the two studies reporting on duodenal taxa, only one further study reported similar taxa in stool. The gastric microbiome was only reported in one study and had taxa similar to the duodenal taxa reported^33^.

#### The gut virome

In addition to bacteria, the gastrointestinal tract also hosts diverse eukaryotic and prokaryotic viruses that make up the virome. The virome is dominated by prokaryotic viruses or bacteriophages^36^. Two studies described associations between the eukaryotic gut virome^30^ and prokaryotic virome and stunting^23,30^. In Desai et al., no viral taxa were associated with growth (stunting) indices, and the viral diversity and abundance were not statistically different between children with adequate and poor growth velocities. They also reported an increase in bacteriophage richness with an increase in bacterial richness in children with adequate and moderate growth, which was not observed in children with poor growth. Similarly, in Mirzei et al., phage diversity and richness were higher in non-stunted children than in stunted children, and phage diversity, parallel with bacterial diversity, was higher in non-stunted children than in stunted children^23^.

#### Metabolic Pathways

Of the studies included, only one study^32^ reported on metagenomic pathways associated with linear growth. The study evaluated the microbiome, including associations with growth in HIV-exposed and non-exposed children in rural Zimbabwe. Although no significant taxonomic differences were observed in stunted and non-stunted children, a spectrum of metabolic pathways associated with and could predict linear growth. They show that pathways encoding lipid biosynthesis, B vitamins, and purine and pyrimidine biosynthesis were predictive of LAZ at 1 and 2 months, while at 3 and 6 months, pathways encoding fermentation and carbohydrate biosynthesis were better predictors of LAZ. At 18 months, carbohydrate and amino acid degradation pathways predicted linear growth.

## Discussion

Childhood stunting has been associated with micronutrient deficiencies, repeated enteric infections that increase both nutrient loss and energy requirements, and helminthic infections^37,38^. We provide a synthesis of recent data from 14 studies that identify microbiota phenotypes associated with stunting across cohorts and geography to better understand how the microbiota may interact with the pathophysiology of stunting. Our results show that all but two studies^31,32^ identified associations between the gut microbiome and childhood stunting. We also show that while some bacterial taxa differed between stunted and non-stunted children, their associations were inconsistent across the studies at both phylum and genus levels. No one genus was associated with stunting across all studies. However, stunting was consistently associated with abundant putative pathobionts, including *Escherichia/Shigella, Campylobacter, Desulfovibrio*, and *Neisseria*. On the other hand, the duodenal microbiota in stunted children was abundant in taxa thought to normally reside in the nasopharyngeal tract. Specific taxonomic categories, including *Veillonela, Neisseria, Streptococcus*, and *Haemophilus*, were reported with an increased frequency in stunted children across geographies in both duodenal and fecal samples.

One possible explanation for observed differences between studies could be variability in the definition of stunting among studies. While the WHO growth standard using Z-scores (HAZ/LAZ<-2) is widely employed to define stunting, some studies defined stunting as a change in Z-scores between two time points. Ongoing discussion exists about the best approach to identify children with growth deficits and stunting. Some researchers argue that children described as ‘not stunted’ by WHO standards could have significant growth deficits and experience stunting even as they fit into the normal growth range and that there is no significant change in their risks and consequences of stunting simply by crossing the stunting cutoff. They suggest that a more sensitive definition of stunting should be based on a child’s growth between two time points^20^. This approach may be more sensitive than cross-sectional measurements of stunting as it takes into account the child’s growth trajectory over time and measures the magnitude of growth faltering.

Our review identified several associations of the gut microbiome that support mechanistic hypotheses about how the gut microbiome may impact childhood stunting. Stunting is a nutritional disorder within the spectrum of malnutrition characterized by distinct clinical and phenotypic sequelae and risk factors^39^. While this review focuses strictly on studies that measure stunting as an outcome, there are potential mechanistic overlaps with the extensive literature on microbiome and malnutrition^40–42^. Gut microbiome immaturity may also contribute to stunting. Several of the taxa identified in this review have also been reported in studies in Bangladesh and Malawi evaluating the microbiome in children suffering from severe acute malnutrition^40,43,44^. In these studies, gut microbiome immaturity was evaluated using machine-learning techniques on age-discriminatory bacterial species and reported as a ‘microbiota for age Z-score’ defining the degree to which a gut microbiota deviates from that of healthy age-matched children—an immature microbiome. The immature microbiome associating with severe malnutrition was characterized by a reduced microbial diversity, a depletion of *Faecalibacterium, Bifidobacterium, Clostridium*, and *Ruminococcus*, and an increase in pathogenic microorganisms at 18 months. The studies also report that diarrhea, formula-feeding and diarrheal events had significant effects on the relative microbiota maturation index. The authors hypothesize that microbiota immaturity can contribute to systemic inflammation, impaired nutrient synthesis, and absorption, exacerbating the malnutrition cycle.

Another shared mechanism is the gut microbiome’s role in maintaining the integrity of the intestinal barrier that holds back microorganisms, antigens, and toxins from the mucosal tissues. A disturbance of the gut microbiome can lead to increased intestinal permeability, which can cause intestinal inflammation. Inflammation, in turn, can alter the structure and function of the small intestinal villi, reducing the absorptive surface area for nutrient uptake and limiting the uptake of essential nutrients^45,46^. Species, particularly from the Pseudomonadota phylum, reported in this review to associate with stunting, have been shown to activate inflammatory cascades ^47,48^ and have been associated with dysbiosis, disease, and malnutrition in children^49,50^. Although only two studies^23,29^ in this review observed significantly higher abundance at the phylum level, at the genus level, consistent negative associations for stunting were observed for putative pathobionts such as *Escherichia/Shigella*^23,24,27,33^, C*ampylobacter*^26,33,34^, *Desulfovibrio*, and *Neisseria*^29,33^ from this phylum. These taxa have also been reported in studies evaluating malnourished children in Bangladesh^51^ and children with Kwashiorkor and Marasmus, a severe form of malnutrition, from Niger and Senegal^49,52^ and The Gambia^53^. These results suggest that some microbiome characteristics are shared across the malnutrition spectrum, with these taxa associating with differing phenotypic/clinical outcomes such as stunting, moderate acute malnutrition (MAM), and more severe phenotypes such as Kwashiorkor and Marasmus. It is unclear if these bacteria result from other underlying processes or are causal in the stunting pathway. However, these pathobionts, particularly in a disturbed microbial ecosystem, have pathogenic potential, with the ability to secrete toxins, invade the intestinal epithelium, and trigger inflammatory responses^47,48,54^. *Desulfovibrio*, for example, plays a role in sulfate reduction by breaking down dietary sulfates, producing hydrogen sulfide, high concentrations of which may cause inflammation and damage to the gut lining^55^, while *Escherichia/Shigella*, which share many virulence strategies, can cause damage to enterocytes, cell death and tissue damage, igniting pro-inflammatory cascades^56,57^ Even without obvious symptoms, this can increase the risk of gastroenteritis and infections. It can also cause a redirection of the limited amino acids and energy needed for growth to immune function, which can worsen growth impairment^58^.

Our review also showed that stunted children often have a lower abundance of putative butyrate producers, including *Faecalibacterium, Megasphera, Blautia*, and *Bifidobacterium*, and an increased abundance of *Ruminococcus*. These taxa are known to produce microbial metabolites known as short-chain acids (SCFAs), which serve as an important source of energy for colonic cells, aid in metabolic processes, and maintain the health of the colonic mucosa by inhibiting the pathways that lead to the production of pro-inflammatory cytokines, thereby regulating mucosal inflammation^59–61^. They also induce innate immunity and play an important role in assimilating nutrients and gut maturation^48,62,63^. The importance of butyrate producers was demonstrated by Blanton et al. when growth and metabolic abnormalities were partially restored to undernourished mice after the addition of R*uminococcus gnavus* and *Clostridium symbiosum*^43^.

Studies using direct biopsy sampling from the duodenum and the stomach provided further insight into the association between the infant gut microbiome and stunting in children. These studies are particularly important as metagenomic samples do not represent all microbial communities found along the gastrointestinal tract mucosa, and the duodenal microbiome would help better understand the ecology in the small intestine where absorption of nutrients occurs^64–66^. In both Vonaesch et al. and Chen et al., a significant overrepresentation of taxa thought to be ordinarily resident in the oropharyngeal tract was observed in the duodenum of stunted children (Fig. 5). Vonaesch et al. phrased their finding as “a decompartmentalization of the gastrointestinal tract,” where microbial taxa, usually resident in the oropharynx, are overrepresented along the gastrointestinal tract^33^, resulting in inflammation and damage of the small intestinal walls, characteristic of environmental enteric dysfunction, leading to stunting. We cross-referenced the duodenal and fecal taxa reported in other studies and observed that other than Chen et al. and Vonaesch et al., only one further study^29^ reported an over-representation of taxa usually found in the nasopharynx in stool samples. These taxa included *Rothia, Veillonella, Streptococcus, Haemophilus, Gemella, and Neisseria* (Fig. 5). However, as no control groups were used for the duodenal studies, it is impossible to tell if these taxa are part of the normal flora whose abundance was altered or if the sampling technique that involves intubation or capsulation could have introduced these bacteria from the oropharynx. Additionally, samples from the oropharyngeal sites were not collected to compare with duodenal samples to determine the similarity of the taxa. Including control groups in such studies remains a challenge, as collecting samples in otherwise healthy children is unethical. To circumvent this bottleneck, there is a need to develop less invasive sample collection techniques in children similar to those used by Shalon et al.^67^.

An analysis of metagenomic pathways suggests that the capacity of the gut microbiome to influence growth depends on the interactions of several metagenomic pathways, and some taxa present in early life may be functionally redundant^32^. B vitamin biosynthesis was consistently a top predictive feature of LAZ, contrasting with results from a large randomized controlled study of vitamin B12 supplementation, which showed that vitamin B-12 levels at baseline did not predict linear growth in those not taking B-12 supplements^15^. Vitamin B12 is both synthesized and utilized by bacteria in the gut microbiome. It may, therefore, simply be a marker of an underlying dysbiotic microbiome and not causal in the linear growth pathway. Conversely, supplementation of vitamin B12 alone in a dysbiotic gut may not redress underlying drivers of dysbiosis, such as inflammation and malabsorption^68^. However, there is very limited data in this area of research, and more data is required to arrive at robust conclusions. Other areas of research have reported promising outcomes for microbiota-directed therapies and can exemplify the methodology required to establish a direct causal link between the gut microbiome and growth outcomes. In their randomized feeding study, where they compared a ready-to-use supplementary food (RUSF) and a microbiota-directed complementary food prototype (MDCF-2) in young children, Gordon’s group was able to demonstrate that modulation of targeted microbiota components found in association studies altered growth outcomes in mouse models and human trials. Children receiving the MDCF-2 showed a change in 21 bacterial taxa significantly positively associated with weight for length zscore and 70 proteins, including mediators of bone growth and neurodevelopment^69^. Similarly, another randomized feeding study by Gehrig et al. 2019, identified an MDCF that was effective in repairing the microbiota, increasing biomarker levels of growth, immune function, and bone formation^70^. Such results support a causal link between microbial patterns and growth outcomes and demonstrate the potential of microbial-directed therapies to improve childhood stunting, and this review provides valuable data on potential target taxa.

### Strengths and limitations

To the best of our knowledge, this is the first systematic review to perform an unbiased synthesis of research on associations between the gut microbiome and childhood stunting in LMICs where the burden of stunting is highest. We used a comprehensive search strategy with a rigorous methodology to limit bias and to provide a comprehensive overview of available data on the gut microbiome and childhood stunting.

Our review has methodological limitations that should be considered when interpreting the results. Firstly, even though all studies were carried out in developing countries, there was an overrepresentation of studies from the Asian region (9, 5, and 2 studies for Asia, Africa, and S. America). Geographic location, diet, sanitation and hygiene, healthcare systems, etc., could impact the synthesis of the results and associations observed across the studies.

In addition to the above-stated, we observed several limitations in the study designs of the included studies. Most studies used small sample sizes, as such, the results cannot be generalized to whole populations. Secondly, cross-sectional studies do not provide sufficient information on the genesis of stunting, while longitudinal studies that utilize sampling with wide time intervals between sample collection points are not ideal for documenting temporal variations and changes in the gut microbiota over time, as age significantly influences the infant gut microbiota^71^.

Third, the studies in this review used 16S rRNA and shotgun sequencing methods. 16S rRNA sequencing methods have been acknowledged to have limited sensitivity and resolution compared to metagenomics^72^. This could be a potential source of the observed discrepant associations, e.g., at the genus level, the alpha diversity (Shannon and Simpson) in this review was lower in studies using Shotgun metagenomics compared to 16S rRNA methods. The different regions of amplification (V4, V3, V1-V3, and V3-V4) and pipelines used (DADA2, QIIME, and Mothur) in the studies in this review could influence the observed results. Additionally, one study used pooled samples for analysis^23^.

Another key limitation was that most studies were observational and only showed associations with stunting but not causality and, as such, did not answer whether a specific gut microbiome composition is a cause or consequence of stunting. However, Chen et al., in their study, evaluated the causal relationship between their findings (that 14 bacterial taxa were associated with poor growth) and the pathogenesis of stunting, providing mechanistic animal and proof of principle data to establish the causal pathway. They did this by administering identified bacterial taxa into germ-free mice. Of the 23 bacterial strains they detected in mice, nine corresponded to the core duodenal bacterial taxa (taxa present in over 80% of duodenal aspirates), which reported a negative correlation with LAZ z-scores showing that stunting phenotypes can be transmitted via microbial taxa^22^. More mechanistic proof of principle, animal, and clinical studies are needed to establish the causal pathway.

We observed high heterogeneity among the studies, which precluded a meta-analysis. Variations in the age of participants (from birth to 5 years), the definition of the outcome, the study design, sequencing techniques used, and geographic location made pooling results difficult. As such, we only provide a narrative synthesis of the data, and caution should be taken when generalizing these findings.

Despite the limitations listed above, this review shows that stunted children can have distinct differences in microbial composition, suggesting a potential role of the gut microbiome in childhood stunting. These findings provide important groundwork for further research to (i) improve our understanding of the mechanisms underlying the gut microbiome’s influence on childhood stunting and (ii) develop targeted and effective interventions to prevent and improve stunting in children.

### Impact, Future Direction, and Conclusion

Understanding the role of the gut microbiome in human health, particularly childhood stunting, has important implications for developing interventions aimed at and improving stunting outcomes. As such, using the gut microbiome to improve health outcomes is a promising area for future research. There is an urgent need for microbiome studies to have clinically relevant results. Our systematic review identified inconsistent phenotype association with stunting and future studies should move this field forward by exploring the mechanisms and functions the microbiome may play in predisposing young children to stunting. Studies should first identify microbial compositional or metabolic profiles that can be used as biomarkers for early diagnosis of stunting. Current studies in this review provide a descriptive characterization of the gut microbiome, which provides valuable information on the composition of bacterial taxa but does not fully capture the mechanistic and functional roles and interactions of these bacteria within the gut ecosystem. More studies combining advanced techniques such as metagenomics, metabolomics, and transcriptomics would provide a more comprehensive view of present organisms, their functions, metabolic pathways, and the changes they induce in the gut to contribute to child growth. Second, future research should identify mechanistic pathways that permit microbiome-based interventions to treat stunting more effectively. Therapies targeting the gut microbiome, such as microbiota-directed foods designed to promote the growth of taxa associated with healthy growth, have been developed as potential alternatives or complements to standard management of stunting^69,70^. However, these approaches need further research to determine their safety and effectiveness and must be easily accessible and affordable in resource-limited settings where the need is greatest.

In conclusion, this systematic review highlights associations between gut microbiome composition and childhood stunting. While there is growing interest in understanding the gut microbiome’s role in childhood stunting, current research remains limited. In this review we show both inconsistent and consistent changes in microbial composition across different studies between stunted and non-stunted children. Future research should employ improved study methodologies evaluating the function of the microbiome in stunted children. Such an evidence base would provide hope that a broader range of microbiome-based interventions may be employed to prevent and reverse the devastating impacts of stunting in children in low- and middle-income settings in the future.

## Methods

We conducted a systematic literature search for studies examining any gut microbiota associations with early childhood growth. Our search strategy followed the Population, Intervention, Comparison, and Outcomes (PICO) and Preferred Reporting Items for Systematic reviews and Meta-analyses (PRISMA) guidelines for reporting systematic reviews^73,74^, and the protocol was registered on PROSPERO (#CRD42022307788). A meta-analysis on alpha diversity was conducted using available raw data to determine the heterogeneity of the studies.

### Eligibility criteria

Studies were included if they used randomized clinical trials (RCTs) or observational study designs. Further inclusion criteria were: studies in under-five children residing in an LMIC who had both their gut microbiome profiled and stunting status or growth velocity documented; studies using high throughput sequencing methods such as 16S rRNA, Shotgun metagenomic sequencing methods, etc., to define the gut microbiome; studies reporting on the presence and abundance of microbial taxa in stunted and healthy children. Studies were excluded if they did not provide sufficient information to investigate the question of interest (i.e., missing sequencing methodology, age of the child, participant stunting status), if they were case reports on single participants, or if they reported on the microbiota after specific interventions without data on baseline status.

### Data Sources and Search Strategy

A systematic literature review was conducted in Global Medicus Index, MEDLINE, and EMBASE to identify published articles and abstracts between 2005 and February 29, 2023. Searches using medical subject headings (MeSH) terms and keywords or combinations of free-text words for “gut microbiota,” “children,” “LMICs,” and stunting” were used in the search strategy Boolean operators (“AND,” “OR,” and “NOT”) were used to broaden or narrow the search results (Supplementary Table 1). Identified articles were exported to Rayyan, where duplicate records were identified and removed. Two reviewers (M.C and V.H) independently reviewed abstracts, and articles meeting inclusion criteria were selected to review the full-text article further. We also examined the reference lists of selected articles to identify additional relevant studies. Any disagreements on the studies to include were resolved through a discussion, and when no consensus was reached, a third reviewer (M.S) was consulted. The search strategy and results are shown in Supplementary Table 1.

### Study selection and data extraction

Study selection was based on the inclusion and exclusion criteria. The first selection was based on titles, and the second was on abstracts of selected titles. Finally, full-text screening was conducted to decide the final articles to be included in the review. Abstracts, titles, and reviews of the full text of selected abstracts were independently done by two reviewers (M.C and V.H). Any disagreements on the studies to include were resolved by consensus. A third reviewer (C.C.L) resolved conflicts if an agreement on inclusion was not reached. Data extraction from the eligible studies was performed independently by M.C. and V.H. using an Excel spreadsheet and compared. The data extracted included the study title, author, year of publication, study objective, location, study design, inclusion and exclusion criteria, stunting definition, study duration, and analytical methods used to describe the gut microbiome, study population, and all reported results on bacterial diversity and richness and bacterial taxonomic composition. Any missing data were noted in the reports. The data extracted were screened for completeness and accuracy by M.C. and V.H.

### Exposures and outcomes

The primary exposure of interest was the gut microbiota, specifically the alpha and beta-diversity and bacterial taxonomic composition (phylum and genus) and metagenomic pathways. The primary outcome for this review was childhood stunting, defined as either a length/height-for-age z-score (LAZ/HAZ) less than two standard deviations below the standard child growth median, or poor growth velocity, defined as a negative change in LAZ/HAZ between two growth measurement points. We therefore sought to compare the microbiome diversity, taxonomic composition, and metagenomic pathways of stunted versus non-stunted children across studies.

### Quality assessment

Quality assessment was conducted on all the studies using the Joanna Briggs Institute (JBI) assessment checklist for case-control, cross-sectional, and cohort studies^75^ by two independent reviewers (M.C. and C.C.L.).

### Supplementary information

Supplemental Material

## Data Availability

All statistical analyses were carried out in R software R 4.1.0. All graphs were plotted using ggplot2 v3.2.1, cowplot v1.1.3, and forestplot v 3.1.3 packages. No customized codes were used in the analyses. The data used to create the figures is available on Figshare (10.6084/m9.figshare.25577259).
