# Supplementary material for: Systematic review of associations between gut microbiome composition and stunting in under-five children
Source: NPJ Biofilms Microbiomes. 2024 May 23;10:46. doi: 10.1038/s41522-024-00517-5 (PMC11116508; doi:10.1038/s41522-024-00517-5)
Supplement: Supplementary file 1 — Supplemental Material [file 41522_2024_517_MOESM1_ESM.pdf]

Supplementary Table 1. Search strategy and search results

**Embase Classic+Embase <1947 to 2023 February 24>**			
Search history sorted by search number ascending			
**#**	**Searches**	**Results**	**Type**
1	Gut bacteria that prevent growth impairments transmitted by microbiota from malnourished children.m_titl.	1	Advanced
2	(Stunting Is Preceded by Intestinal Mucosal Damage and Microbiome Changes and Is Associated with Systemic Inflammation in a Cohort of Peruvian Infants).m_titl.	1	Advanced
3	(Growth velocity in children with Environmental Enteric Dysfunction is associated with specific bacterial and viral taxa of the gastrointestinal tract in Malawian children).m_titl.	1	Advanced
4	1 or 2 or 3	3	Advanced
5	((developing country/ or low income country/ or middle income country/ or (low* income* adj3 (countr* or nation* or economy or economies)).tw,kw. or (middle income* adj3 (countr* or nation* or economy or economies)).tw,kw. or (low* middle adj3 (countr* or nation* or economy or economies)).tw,kw. or (LMIC or LMICs).tw,kw. or ((LIC or LICs) adj3 (countr* or nation* or economy or economies)).tw,kw. or "transition* countr*".tw,kw. or ((underserved or "under served" or deprived or poor*) adj3 (country or countries or nation? or economy or economies)).tw,kw. or ((Developing or "under developed" or underdeveloped or "less* developed" or "third world") adj3 (country or countries or nation? or economy or economies)).tw,kw. or ((Developing or "under developed" or underdeveloped or "less* developed") adj2 world).tw,kw. or ((Africa* not "African American*") or (Asia* not "Asian American*")).ti,ab,in,ad,kw. or (Afghanistan* or Albania* or Algeria* or Angola* or Anguilla* or Antigua or Barbuda or Argentin* or Armenia* or Aruba* or Azerbaijan* or Bahamas or Bahrain* or Bangladesh* or Barbados or Belarus* or Belize* or Benin* or Bermuda* or Bhutan* or Bolivia* or Bosnia* or Herzegovina or Borneo or Botswana* or Brazil* or Brunei* or Bulgaria* or "Burkina Faso" or Burundi*).ti,ab,in,ad,kw. or (Cambodia* or Cameroon* or "Cape Verde*" or "Cabo Verde*" or Caribbean* or "Cayman Is*" or Chad or Chile* or China or Chinese or (Colombia* not "British Colombia*") or Comoros or Congo or "Cook Island*" or "Costa Rica*" or "ivory coast" or "cote d'ivoire" or Croat* or Cuba* or Cyprus or Cypriot* or Czech* or Djibouti* or Dominica*).ti,ab,in,ad,kw. or (Ecuador* or Egypt* or "El Salvador" or Eritrea* or Estonia* or Ethiopia* or "Falklands Is*" or Fiji* or Gabon* or Gambia* or Ghana* or Gibralta* or Grenada* or Guatemala* or Guinea* or Guiana* or Guyana* or Haiti* or Hondura* or "Hong Kong*" or Hungary or Hungarian* or India or (Indian? not "American Indian?") or Indonesia* or Iran* or Iraq* or Israel*).ti,ab,in,ad,kw. or (Jamaica* or Jordan* or Kazakhstan* or Kenya* or Kiribati* or Korea* or DPRK or Kosovo* or Kuwait* or Kyrgyz* or "Lao PDR" or "Lao People*" or Laos or Laotian or Latvia* or Lebanon or Lebanese or Lesotho or Liberia* or Libya* or Lithuania*).ti,ab,in,ad,kw. or (Macao* or Macau or Macedonia* or Madagasca* or Malawi* or Malaysia* or Maldives or Mali or Malta or	12328175	Advanced

	Maltese or "Marshall Islands" or Mauritania* or Mauritius or Mayotte* or Melanesia* or Mexico or Mexican? or Micronesia* or Moldova* or Mongolia* or Montenegro* or Morocco or Moroccan? or Mozambique* or Myanmar*).ti,ab,in,ad,kw. or (Namibia* or Nauru* or Niue* or Nepal* or "Netherlands Antilles*" or "Dutch Antilles" or "New Caledonia*" or Nicaragua* or Niger or Nigeria* or Oman* or Pakistan* or Palau* or Palestin* or Panama or "Papua New Guinea*" or Paraguay or Peru* or Peruvian* or Philippines* or Pilipin* or Filipin* or Poland or Polish or Polynesia* or Qatar* or Romania* or Russia* or Rwanda*).ti,ab,in,ad,kw. or (Samoa* or "Sao Tome*" or Principe* or Saudi or Senegal* or Serbia* or Seychelles or "Sierra Leone" or Singapor* or Slovak* or Sloven* or "Solomon Islands" or Somalia* or "Sri Lanka*" or "S* Kitts and Nevis" or "S* Lucia" or "S* Helena" or "S* Vincent and the Grenadines" or "South America*" or Sudan* or Suriname* or Swaziland* or Syria*).ti,ab,in,ad,kw. or (Taiwan* or Taipei* or Tajikistan* or Tanzania* or Thai* or Timor* or Tobago or Togo or Tokelau or Tonga or Trinidad or Tunisia* or Turkey or Turkish or Turkmenistan* or "Turks and Caicos" or Tuvalu* or Uganda* or Ukrain* or "United Arab Emirates" or Uruguay* or Uzbekistan* or Vanuatu* or Venezuela* or Vietnam* or "Virgin Is*" or "Wallis and Futuna" or Futuna or "West Bank" or Gaza or Yemen* or Zambia* or Zimbabw*).ti,ab,in,ad,kw. or exp Africa/ or caribbean islands/ or "anguilla (country)"/ or "antigua and barbuda"/ or aruba/ or bahamas/ or barbados/ or caribbean netherlands/ or cayman islands/ or cuba/ or dominica/ or dominican republic/ or grenada/ or haiti/ or jamaica/ or netherlands antilles/ or "saint kitts and nevis"/ or saint lucia/ or "saint vincent and the grenadines"/ or "trinidad and tobago"/ or "turks and caicos islands"/ or "virgin islands (british)"/ or "virgin islands (u.s.)"/ or bermuda/ or "falkland islands (malvinas)"/ or saint helena/ or "sao tome and principe"/ or exp "South and Central America"/ or exp Mexico/ or asia/ or kazakhstan/ or kyrgyzstan/ or exp middle east/ or exp south asia/ or tajikistan/ or turkmenistan/ or uzbekistan/ or far east/ or exp china/ or korea/ or mongolia/ or philippines/ or exp southeast asia/ or taiwan/ or exp pacific islands/ or exp indian ocean/ or exp Eastern Europe/ or gibraltar/ or malta/ or Georgia.ti,ab.) not "georgia (u.s.)"/) or (Montserrat not (Spain or Espana)).ti,ab,in,ad,kw.		
6	exp growth disorder/ or ((growth adj2 (disorder* or velocity or faltering or deficit or stunted or failure or retardation or alteration or disturbance or trajectory or linear)) or stunt* or (undernutrition or malnutrition or nutrition) or HAZ or LAZ or (height adj2 age) or (length adj2 age)).ti,ab,kf.	598601	Advanced
7	exp intestine flora/ or (((gut or gastrointestinal or gastric) adj3 (microbiome* or microbiota* or health or flora or microflora or microbial)) or bacterial-flora or Enteric-Bacteria).ti,ab,kf.	128813	Advanced
8	child/ or abandoned child/ or adopted child/ or hospitalized child/ or exp infant/ or institutionalized child/ or preschool child/ or toddler/ or (child* or infan* or neonatal).ti,ab,kf.	4175127	Advanced
9	5 and 6 and 7 and 8	764	Advanced
10	limit 9 to (conference abstracts or embase)	**649**	

Ovid MEDLINE(R) ALL <1946 to February 24, 2023>

Search history sorted by search number ascending

#	Searches	Results	Type
1	Developing Countries/ or (low* income* adj3 (countr* or nation* or economy or economies)).tw,kf. or (middle income* adj3 (countr* or nation* or economy or economies)).tw,kf. or (low* middle adj3 (countr* or nation* or economy or economies)).tw,kf. or (LMIC or LMICs).tw,kf. or ((LIC or LICs) adj3 (countr* or nation* or economy or economies)).tw,kf. or "transition* countr*".tw,kf. or ((underserved or "under served" or deprived or poor*) adj3 (country or countries or nation? or economy or economies)).tw,kf. or ((Developing or "under developed" or underdeveloped or "less* developed" or "third world") adj3 (country or countries or nation? or economy or economies)).tw,kf. or ((Developing or "under developed" or underdeveloped or "less* developed") adj2 world).tw,kf. or ((Africa* not "African American*") or (Asia* not "Asian American*")).ti,ab,in,kf. or (Afghanistan* or Albania* or Algeria* or Angola* or Anguilla* or Antigua or Barbuda or Argentin* or Armenia* or Aruba* or Azerbaijan* or Bahamas or Bahrain* or Bangladesh* or Barbados or Belarus* or Belize* or Benin* or Bermuda* or Bhutan* or Bolivia* or Bosnia* or Herzegovina or Borneo or Botswana* or Brazil* or Brunei* or Bulgaria* or "Burkina Faso" or Burma or Burmese or Burundi*).ti,ab,in,kf. or (Cambodia* or Cameroon* or "Cape Verde*" or "Cabo Verde*" or Caribbean* or "Cayman Is*" or Chad or Chile* or China or Chinese or (Colombia* not "British Colombia*") or Comoros or Congo or "Cook Island*" or "Costa Rica*" or "ivory coast" or "cote d'ivoire" or Croat* or Cuba* or Cyprus or Cypriot* or Czech* or Djibouti* or Dominica*).ti,ab,in,kf. or (Ecuador* or Egypt* or "El Salvador" or Eritrea* or Estonia* or Ethiopia* or "Falklands Is*" or Fiji* or Gabon* or Gambia* or Ghana* or Gibralta* or Grenada* or Guatemala* or Guinea* or Guiana* or Guyana* or Haiti* or Hondura* or "Hong Kong*" or Hungary or Hungarian* or India or (Indian? not "American Indian?") or Indonesia* or Iran* or Iraq* or Israel*).ti,ab,in,kf. or (Jamaica* or Jordan* or Kazakhstan* or Kenya* or Kiribati* or Korea* or DPRK or Kosovo* or Kuwait* or Kyrgyz* or "Lao PDR" or "Lao People*" or Laos or Laotian or Latvia* or Lebanon or Lebanese or Lesotho or Liberia* or Libya* or Lithuania*).ti,ab,in,kf. or (Macao* or Macau or Macedonia* or Madagasca* or Malawi* or Malaysia* or Maldives or Mali or Malta or Maltese or "Marshall Islands" or Mauritania* or Mauritius or Mayotte* or Melanesia* or Mexico or Mexican? or Micronesia* or Moldova* or Mongolia* or Montenegro* or Morocco or Moroccan? or Mozambique* or Myanmar*).ti,ab,in,kf. or (Namibia* or Nauru* or Niue* or Nepal* or "Netherlands Antilles*" or "Dutch Antilles" or "New Caledonia*" or Nicaragua* or Niger or Nigeria* or Oman* or Pakistan* or Palau* or Palestin* or Panama or "Papua New Guinea*" or Paraguay or Peru* or Philippines* or Pilipin* or Filipin* or Poland or Polish or Polynesia* or Qatar* or Romania* or Russia* or Rwanda*).ti,ab,in,kf. or (Samoa* or "Sao Tome*" or Principe* or Saudi or Senegal* or Serbia* or Seychelles or "Sierra Leone" or Singapor* or Slovak* or Sloven* or "Solomon Islands" or Somalia* or "Sri Lanka*" or "S* Kitts and Nevis" or "S* Lucia" or "S* Helena" or "S* Vincent and the Grenadines" or "South America*" or Sudan* or Suriname* or Swaziland* or Eswantini or Syria*).ti,ab,in,kf. or (Taiwan* or Taipei* or Tajikistan* or Tanzania* or Thai* or Timor* or Tobago or Togo or Tokelau or Tonga* or Trinidad or Tunisia* or Turkey or Turkish or Turkmenistan* or "Turks and Caicos" or Tuvalu* or Uganda* or Ukrain* or "United Arab Emirates" or Uruguay* or Uzbek* or Vanuatu* or Venezuela* or Vietnam* or "Virgin Is*" or "Wallis and Futuna" or Futuna or "West Bank" or Gaza or Yemen* or Zambia* or Zimbabw*).ti,ab,in,kf. or exp Africa/ or west indies/ or "antigua and barbuda"/ or bahamas/ or barbados/ or "british virgin islands"/ or cuba/ or dominica/ or dominican republic/ or grenada/ or haiti/ or jamaica/ or saint lucia/ or "saint vincent and the grenadines"/ or "saint kitts and nevis"/ or "trinidad and tobago"/ or central america/ or costa rica/ or el salvador/ or guatemala/ or honduras/ or nicaragua/ or exp panama/ or mexico/ or exp south america/ or exp Atlantic Islands/ or asia/ or exp asia, central/ or exp asia, southeastern/ or exp asia, western/ or far east/ or exp china/ or exp korea/ or Taiwan/ or exp Middle East/ or pacific islands/ or exp melanesia/ or micronesia/ or palau/ or polynesia/ or exp samoa/ or tonga/ or exp Europe, Eastern/ or Cyprus/ or Malta/ or Gibraltar/ or (Georgia.ti,ab. not Georgia/) or (Montserrat not (Spain or Espana)).ti,ab.	8637672	Advanced

2	exp growth disorder/ or ((growth adj2 (disorder* or velocity or faltering or deficit or stunted or failure or retardation or alteration or disturbance or trajectory or linear)) or stunt* or (undernutrition or malnutrition or nutrition) or HAZ or LAZ or (height adj2 age) or (length adj2 age)).ti,ab,kf.	337514	Advanced
3	exp "gastrointestinal microbiome"/ or (((gut or gastrointestinal or gastric) adj3 (microbiome* or microbiota* or health or flora or microflora or microbial)) or bacterial-flora or Enteric-Bacteria).ti,ab,kf.	77306	Advanced
4	exp child/ or "Child, preschool"/ or Infant/ or "Infant, newborn"/ or "Infant, low birth weight"/ or "Infant, small for gestational age"/ or "Infant, very low birth weight"/ or "Infant, postmature"/ or "Infant, premature"/ or Minors/ or "Child hospitalized"/ or "Child institutionalized"/ or "Disabled children"/ or Pediatrics/ or (child* or infan* or neonatal).ti,ab,kf.	3400340	Advanced
5	1 and 2 and 3 and 4	**434**	
**Global Index Medicus (1986-2023)**			
1	tw:(tw:((growth OR stunt* OR undernutrition OR malnutrition OR nutrition OR haz OR laz OR "height for age" OR "length for age") AND (microbiome OR microbiota OR microflora) AND (child OR children OR juvenile OR neonat* OR infant*)))	113	
**SCOPUS**			
1	(TITLE-ABS-KEY ("HAZ" OR "LAZ" OR "height for age" OR "length for age" OR "malnutrition" OR "undernutrition" OR "growth" OR "stunt*") AND TITLE-ABS-KEY ("gut microbio*" OR "microflora" OR "intestinal microbio*") AND TITLE-ABS-KEY ("LMIC" OR "developing countr*"))	120	

**Summary of searches**	
Medline	434
Embase	649
Global Index Medicus	113
SCOPUS	120
Total	1316

Supplementary Table 2. Quality assessment using the Joanna Briggs Institute (JBI) Critical Appraisal Checklist for Cohort, case-control, and cross-sectional studies

a) Summary of quality assessments (cohort studies)

**JBI Checklist /Study**	1. Were the two groups similar and recruited from the same population ?	2.Were the exposures measured similarly to assign people to both exposed and unexposed groups?	3. Was the exposure measured in a valid and reliable way?	4. Were confounding factors identified?	5. Were strategies to deal with confounding factors stated?	6*. Were the groups/participants free of the outcome at the start of the study (or at the moment of exposure)?	7. Were the outcomes measured in a valid and reliable way?	8. Was the follow up time reported and sufficient to be long enough for outcomes to occur?	9. Was follow up complete, and if not, were the reasons to loss to follow up described and explored?	10. Were strategies to address incomplete follow up utilized?	11. Was appropriate statistical analysis used?	Score (%)	Risk
Gough et al., 2015	Y	Y	Y	X	X	N/A	Y	Y	Y	N/A	Y	7 (70.0)	Moderate
Desai et.al., 2020	Y	Y	Y	Y	Y	N/A	Y	Y	X	X	Y	8 (80.0)	Low
Kamng'ona et al., 2019	Y	Y	Y	X	X	N/A	Y	Y	X	X	Y	6 (60)	Moderate
Rouhani et. al., 2020	Y	Y	Y	Y	Y	N/A	Y	Y	Y	N/A	Y	9 (90.0)	Low
Zambruni, et al., 2019	Y	Y	Y	X	X	N/A	Y	Y	Y	N/A	Y	7 (70.0)	Moderate
Robertson et al., 2023	Y	Y	Y	Y	Y	N/A	Y	Y	Y	N/A	Y	9 (90)	Low

*item number 6 was not applicable as it was not relevant for participants to be free of the outcome at baseline

b) Summary of quality assessments (case-control studies)

**JBI Checklist /Study**	1. Were the groups comparable other than the presence of disease in cases or the absence	2. Were cases and controls matched appropriately?	3. Were the same criteria used for identification of cases and controls?	4. Was exposure measured in a standard, valid and reliable way?	5. Was exposure measured in the same way for cases and controls?	6. Were confounding factors identified?	7. Were strategies to deal with confounding factors stated?	8. Were outcomes assessed in a standard, valid and reliable way for cases and controls?	9. Was the exposure period of interest long enough to be meaningful?	10. Was appropriate statistical analysis used?	Score (%)	Risk

	of disease in controls?											
Khan Mirzaei et al., 2020	Y	N	X	Y	Y	Y	X	X	Y	Y	6 (60.0)	Moderate
Masrul et al., 2020	Y	X	X	Y	Y	X	X	Y	Y	Y	6 (60.0)	Moderate
Chen et al., 2020	Y	Y	Y	Y	Y	X	X	Y	Y	Y	8 (80.0)	Low
Perin et al., 2020	Y	Y	Y	Y	Y	Y	Y	Y	Y	Y	10 (100)	Low
Dinh et al., 2016	Y	Y	Y	Y	Y	X	X	Y	Y	Y	8 (80.0)	Low

c. Summary of quality assessments (cross-sectional studies) using the JBI appraisal checklist

**JBI Checklist /Study**	1.Were the criteria for inclusion in the sample clearly defined?	2. Were the study subjects and the setting described in detail?	3. Was the exposure measured in a valid and reliable way?	4. Were objective, standard criteria used for measurement of the condition?	5. Were confounding factors identified?	6. Were strategies to deal with confounding factors stated?	7. Were the outcomes measured in a valid and reliable way?	8. Was appropriate statistical analysis used?	Overall *	Risk
Vonaesch et al., 2018	Y	Y	Y	Y	N	N	Y	Y	6 (75.0)	Low
Surono et al., 2021	Y	Y	Y	Y	N	N	Y	Y	6 (75.0)	Low
Shivakumar et al.,2021	Y	Y	Y	Y	X	X	Y	Y	6 (75.0)	Low

Abbreviations: Y = Yes; N = No; U = Unclear; NA = Not Applicable; JBI: Joanna Briggs Institute

Criteria used to rank the risk of bias:

≤49%=high risk of bias

50-69%=Moderate risk of bias

above 70% = low risk of Bias

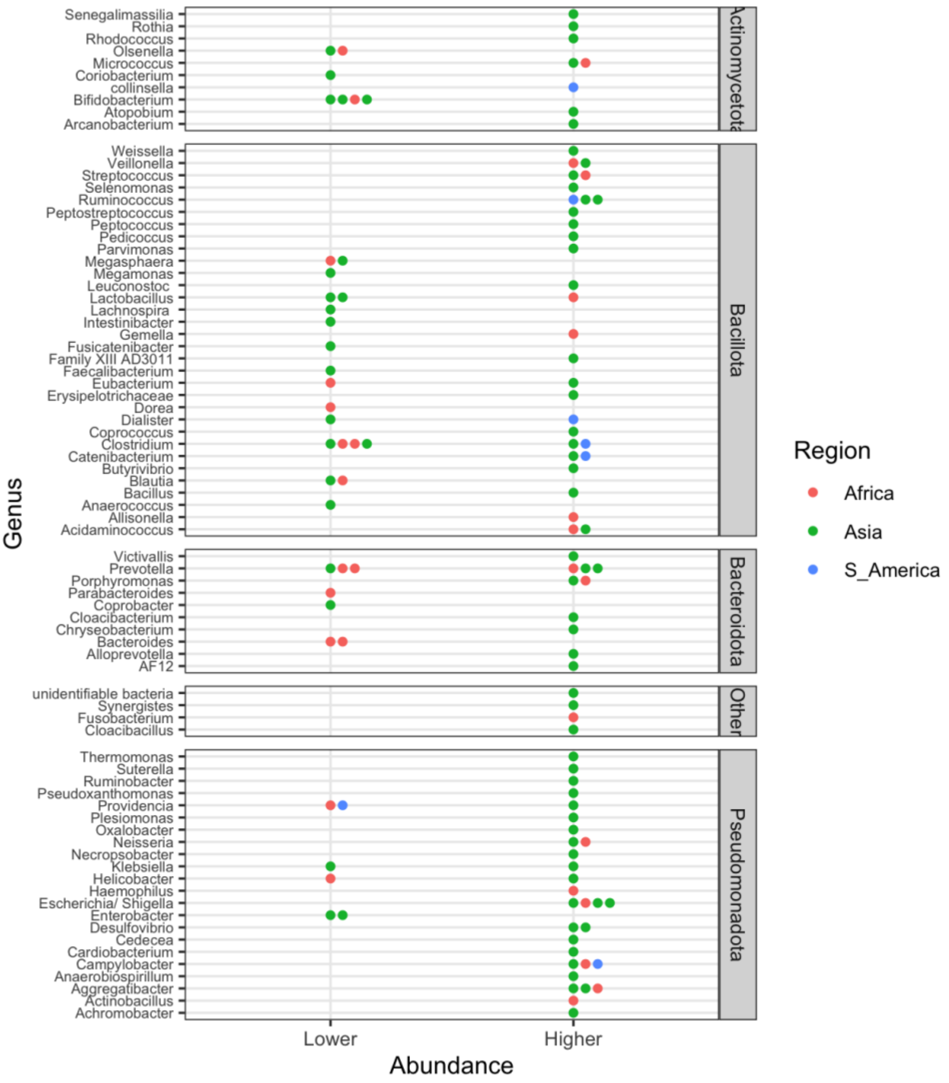

Sup. fig. 1. Bacterial genera with significant differences in relative abundance between stunted and non-stunted children in fecal samples stratified by geographic region. “Other phyla” includes Fusobacteriota, Verrucomicrobiota, and Synergistota.
